# mBAT: a newly developed mobile application for self-screening of pediatric bleeding disorders – a multi-center study

**DOI:** 10.1007/s00277-024-06178-w

**Published:** 2025-01-15

**Authors:** Arpatsorn Sermcheep, Rungrote Natesirinilkul, Patcharee Komvilaisak, Natsaruth Songthawee, Kamala Laohverapanich, Duantida Songdej, Pakawan Wongwerawattanakoon, Praguywan Kadegasem, Ampaiwan Chuansumrit, Nongnuch Sirachainan

**Affiliations:** 1 Division of Hematology and Oncology, Department of Pediatrics, Faculty of Medicine Ramathibodi Hospital, Mahidol University, 270 Rama VI Road, Ratchathewi District, Bangkok, Thailand; 2 Department of Pediatrics, Faculty of Medicine Chiang Mai University Hospital, Chiang Mai, Thailand; 3 Department of Pediatrics, Faculty of Medicine, Srinagarind Hospital, Khon Kaen, Thailand; 4 Department of Pediatrics, Faculty of Medicine, Prince of Songkla University, Songkhla, Thailand

**Keywords:** Bleeding assessment tool, Children, Mobile application, Bleeding disorders, Von Willebrand disease

## Abstract

Bleeding assessment tools (BATs) are used by trained medical personnel for screening bleeding disorders on a one-to-one basis with patients; hence, they are time-consuming and limited in use for large-population screening. The aims of the study were to develop, validate, and demonstrate a Thai BAT mobile application (mBAT) for self-screening of bleeding disorders. mBAT was developed and validated using the paper-based Thai version of pediatric bleeding questionnaire (TPBQ). Then, mBAT was applied to 916 subjects from communities and hematology clinics. Those with scores of ≥3 consented to further investigation of bleeding disorders. mBAT was developed for Thai children using simple sentences, a video introduction, pictures, and auto-calculation. It showed a high correlation with the TPBQ: r = 0.96 and 0.97 in the PBQ and ISTH scoring systems, respectively (P<0.01). A score ≥3 was considered abnormal. The mBAT was applied to 916 subjects, with a mean (range) age of 10.5 (0.07-18.0) years. Most subjects (97.3%) were from the community, and the rest (2.7%) were from hematology clinics. The results showed 41 subjects had abnormal scores, and 35 (16 from the community and 19 from clinics) consented to diagnostic investigation. Eleven subjects (31.4%) were found to have bleeding disorders, including four with VWD, two each with hemophilia A and thrombocytopenia, and one each with factor VII deficiency, platelet dysfunction, and MYH9-related disorder. Thus, the prevalence rates of bleeding disorders in the subjects whose scores ≥3 from community and hematology clinics were 6.25% and 52.6%, respectively. In conclusions, the mBAT had a high correlation with the TPBQ. As a self-screening tool, it could enhance the diagnosis of bleeding disorders.

**Supplementary Information:**

The online version contains supplementary material available at 10.1007/s00277-024-06178-w.

## Introduction

Mild bleeding disorders, such as Von Willebrand disease (VWD), mild hemophilia, and inherited platelet disorders, are usually under-diagnosed because of their minor bleeding symptoms, i.e., epistaxis, bruising, heavy menstrual bleeding, and prolonged bleeding after surgery. The prevalence of VWD in the general population varies between 0.6–1.3%, which is higher than that of hemophilia at 0.002–0.01% of population [1–3]. Furthermore, the estimated prevalence of inherited platelet disorders and rare bleeding disorders is 0.3% and 0.002%, respectively [4]. The under-diagnosed mild bleeding disorders were demonstrated in the report of the World Federation of Hemophilia (WFH) Global Survey with a total of 429,232 patients: hemophilia was the most common, consisting of 59.8% (n = 256,840), followed by VWD at 22.9% (n = 98,293), and other bleeding disorders at 17.3% (n = 74,099) [1]. Similar results were found in the Thailand National Survey Study Report of 2,190 patients, with hemophilia the most common disorder at 88.4% (n = 1,936), followed by VWD at 7.4% (n = 163) and other bleeding disorders at 4.1% (n = 91) [1].

To enhance the diagnosis, bleeding assessment tools (BATs) have been developed for the standardized evaluation of bleeding symptoms. In children, both the Pediatric Bleeding Questionnaire (PBQ) and the International Society on Thrombosis and Hemostasis Bleeding Assessment Tool (ISTH-BAT) have been used for screening patients who present with bleeding problems [5–11]. A previous study showed that BATs can differentiate patients with bleeding disorders from healthy controls. In addition, higher scores were associated with the severity of diseases. The effectiveness of PBQ was confirmed with high sensitivity and specificity for VWD diagnosis at 83% and 79%, respectively. The score ≥2 had a likelihood ratio of 3.9 (95%CI 2.4–6.3) for the diagnosis of VWD [8, 9]. The cut-off score was different for the ISTH-BAT, showing that a score ≥3 in children was determined as abnormal [10, 11].

However, the original English language questionnaire could be limited in its application in countries with different languages. In addition, cultural circumstances might greatly affect the report of bleeding experiences. For example, hypermenorrhea could be considered normal in some cultures; and as a result, the symptom is not reported. Therefore, the original English questionnaire should be translated into the native language of a non-English-speaking country and validated before its implementation.

In 2017, a Thai version of Pediatric Bleeding Questionnaire (TPBQ) was developed by translating the English questionnaires and the scoring keys of the PBQ and ISTH-BAT into Thai language. For validation, it was first applied to children with known bleeding disorders and compared with healthy controls. A score ≥3 on both systems suggested bleeding disorders, including VWD, platelet disorders, and mild hemophilia [12]. For community screening, TPBQ was applied by pediatricians to 309 students. The results demonstrated that eight students had scores of ≥3, and two of them were each found to have VWD types 2A and 1. Therefore, the prevalence of bleeding disorders was 0.65% [13]. In addition, the screening required medical personnel; hence, its use in a large-scale operation was limited.

In 2021, Kaur et al. developed and evaluated an electronic self-administered bleeding assessment tool (eBAT) through a tablet or smart device, used in subjects with bleeding symptoms, a family history of bleeding disorders, or abnormal coagulation labs. The eBAT showed a strong correlation with physician-administered bleeding questionnaires with high sensitivity of 94% for recognized bleeding disorders, and 72% of participants completed the questionnaire in less than 10 minutes [14]. The study opened up the possibility of developing self-screening BAT for the pediatric population. Moreover, the study of self-screening bleeding disorder in the community was limited. Thus, the objectives of this study were to develop and validate a Thai Bleeding Assessment Tool mobile application (mBAT), and to demonstrate its use as a self-screening tool for diagnosis of bleeding disorders in the Thai pediatric population.

## Materials and methods

**Fig. 1:**
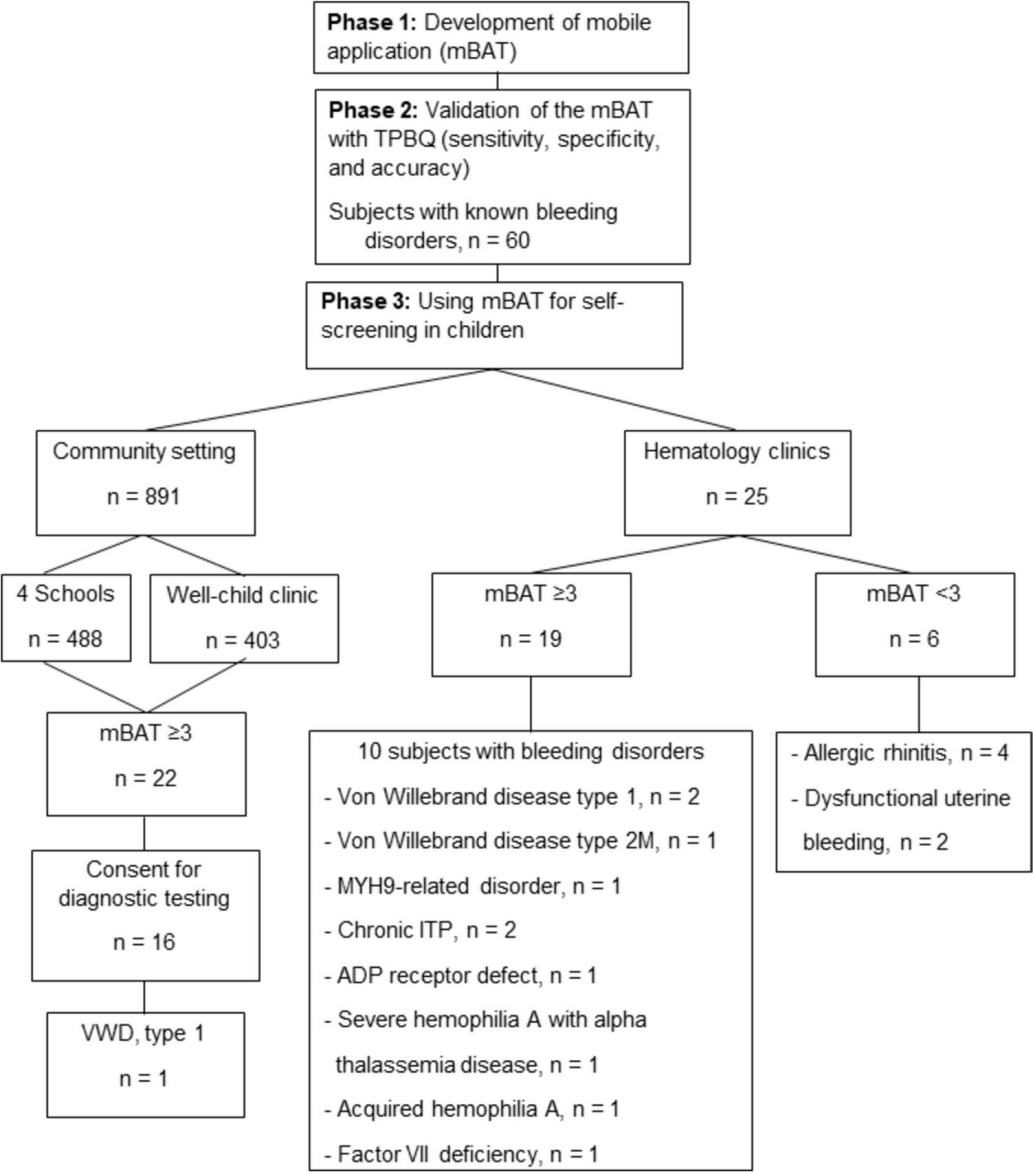
Study flow chart. ITP, immune thrombocytopenia; mBAT, ฺBleeding assessment tool mobile application; TPBQ, Thai version of Pediatric Bleeding Questionnaire

A multi-center cross-sectional study comprised four regional tertiary care hospitals in Thailand: Ramathibodi (Central Region), Chiang Mai University (Northern Region), Srinagarind (North-Eastern Region), and Songklanagarind (Southern Region). The study was divided into three phases: 1) development of mBAT, 2) validation of the mBAT, and 3) using mBAT for self-screening in children. A cross-sectional study was conducted from January 2019 to December 2022 (Fig. 1). Part of the study period was during the COVID-19 pandemic; therefore, correspondence between the study team and the subjects and/their parents to provide knowledge and obtain consent happened in various forms (e.g., dialogue, paper sheets, video clips) and channels (e.g.; at school, by post, LINE – a freeware app and service for instant messaging and social networking). Consent was obtained from all subjects and normal controls for the study; for < 7-year-old subjects, consent was from their parents; otherwise, consent was from both the subjects and their parents. Parents were advised to answer the questionnaire with the subjects because subjects may not recognize bleeding symptoms in early life. The study was approved by the hospital's Committee on Human Rights Related to Research Involving Human Subjects (COA. MURA2018/271).

### Phase 1. Development of mBAT

The TPBQ for medical personnel was modified to create a simpler and more understandable self-screening tool for bleeding disorders in children. To enable self-screening, we developed a mobile version of the tool called mBAT, which can be easily downloaded from online platforms such as iOS and Android onto a smartphone or tablet. The study team worked closely with the programmer and creative graphic designer (developing team) in each step of development. The designer is a person with severe hemophilia A and has served as vice president of the Thai Hemophilia Patient Club. Members of the developing team all agree with the designed pictures, words, and sentences.

### Phase 2. Validation of the mBAT

The 2% prevalence of mild bleeding disorders for the calculation of sample size in the validation of mBAT was taken from the summary of prevalences of VWD, inherited platelet disorder, hemophilia, other rare bleeding disorders, and immune thrombocytopenia (ITP) [1–3, 15, 16]. Using the estimate one proportion method, the error (d) was 1%, and the type I error (α) was 5%. As a result, the sample size was estimated to be 60. Therefore, mBAT was applied to 60 subjects (patients at the Hematology Clinic, Ramathibodi Hospital) aged <18 years diagnosed with known bleeding disorders. After being informed, subjects and parents were first asked to complete mBAT or TPBQ. At least two weeks after completing one of the questionnaires (either the mBAT or TPBQ), subjects and parents were scheduled to visit the clinic for the other questionnaire. Subjects or parents could install mBAT onto their smartphones and log in with the provided usernames and passcodes. Subjects and/or parents selected bleeding symptom categories and answered questions regarding their severity and treatment. When the questionnaire was completed, subjects and parents were asked to complete a satisfaction questionnaire using the system usability scale (SUS) [17, 18]. For subjects aged <7 years, the satisfaction questionnaire was performed by parents. At least two weeks after completing the mBAT, subjects and parents had an appointment at the clinic and were interviewed by a physician in the study team using the TPBQ. Scoring results from mBAT and TPBQ were analyzed and compared using correlation coefficient methods. The cut-off bleeding scores of mBAT were performed to determine the sensitivity, specificity, and accuracy of mBAT compared to TPBQ.

### Phase 3. Using mBAT for self-screening in children

A cross-sectional, multi-center study was conducted from September 2020 to December 2022 at four regional tertiary care hospitals: Ramathibodi, Chiang Mai University, Srinagarind, and Songklanagarind. The mBAT was applied to two groups of subjects: community group and hematology clinics. The community group included students (grades 7 to 12) from four secondary schools, ranging in age 12–18 years, and well-child clinics included children ranging in age from 1 month to 18 years visiting the four hospitals’ well-child clinics for vaccinations. All subjects had never been diagnosed with any bleeding disorder. The subjects of the hematology clinics group were 25 patients presenting with bleeding symptoms or referred to outpatient hematology clinics at the Ramathibodi Hospital to diagnose bleeding disorders. Subjects or parents installed the mBAT on their smartphones, logged in, and filled out the questionnaire using the username and password that were individually provided. The study team and video introduction instructed subjects to choose any bleeding symptoms they had ever experienced for accurate bleeding scores, even if it was a minor symptom. Subjects/parents were allowed to change their answers within 72 hours. Subjects with a score of ≥3 were contacted and interviewed by the study team using TPBQ to recheck bleeding symptoms and receive consent to blood tests.

### Laboratory measurements

Blood samples from subjects having a score of ≥3 were tested for a complete blood count (CBC, BC-6800 Plus, Mindray, Shenzhen, China). If the platelet count was normal, the samples were further tested as follows: platelet function analysis-100 (PFA-100, Siemens, Malvern, PA), prothrombin time (PT, Thromborel S®, Sysmex CS-2500, Sysmex, Kobe, Japan), activated partial thromboplastin time (aPTT, Actin FS®, Sysmex CS-2500, Sysmex, Kobe, Japan), thrombin time (TT, Thromboclotin®, Sysmex CS-2500, Sysmex, Kobe, Japan), fibrinogen (Dade thrombin®, Sysmex CS-2500, Sysmex, Kobe, Japan), platelet aggregation test with ADP, collagen, adrenaline, and ristocetin agonist (Revohem®, Helena AggRAM, Helena laboratories, Beaumont, Texas, US), VWF:Activity (INNOVANCE® VWF Ac Assay, Sysmex CS2000i, Bangkok, Thailand), and factor VIII activity (Clotting assay, CS-1600 Sysmex, Kobe, Japan). The additional tests, such as VWF multimer, other specific assays, and genetic studies, were performed according to the results of the aforementioned tests.

### Statistical analysis

Statistical analysis was performed using SPSS software (version 18.0). Descriptive statistics, including the median, were calculated for continuous variables, while categorical variables, such as age, gender, and diagnosis, were summarized as frequencies and percentages. Diagnostic performance was assessed through sensitivity, specificity, accuracy, prevalence, and receiver operating characteristic (ROC) curve analysis, with the area under the curve (AUC) used to determine the optimal cut-off bleeding scores for mBAT. For the validation of mBAT, the sample size was calculated using the estimated one-proportion method, with an allowable error (d) of 1%. Correlation analysis to compare bleeding scores between mBAT and TPBQ was conducted using Pearson’s correlation coefficient. Statistical significance was set at a P-value of <0.05.

## Results

### Phase 1. Development of mBAT

**Fig. 2:**
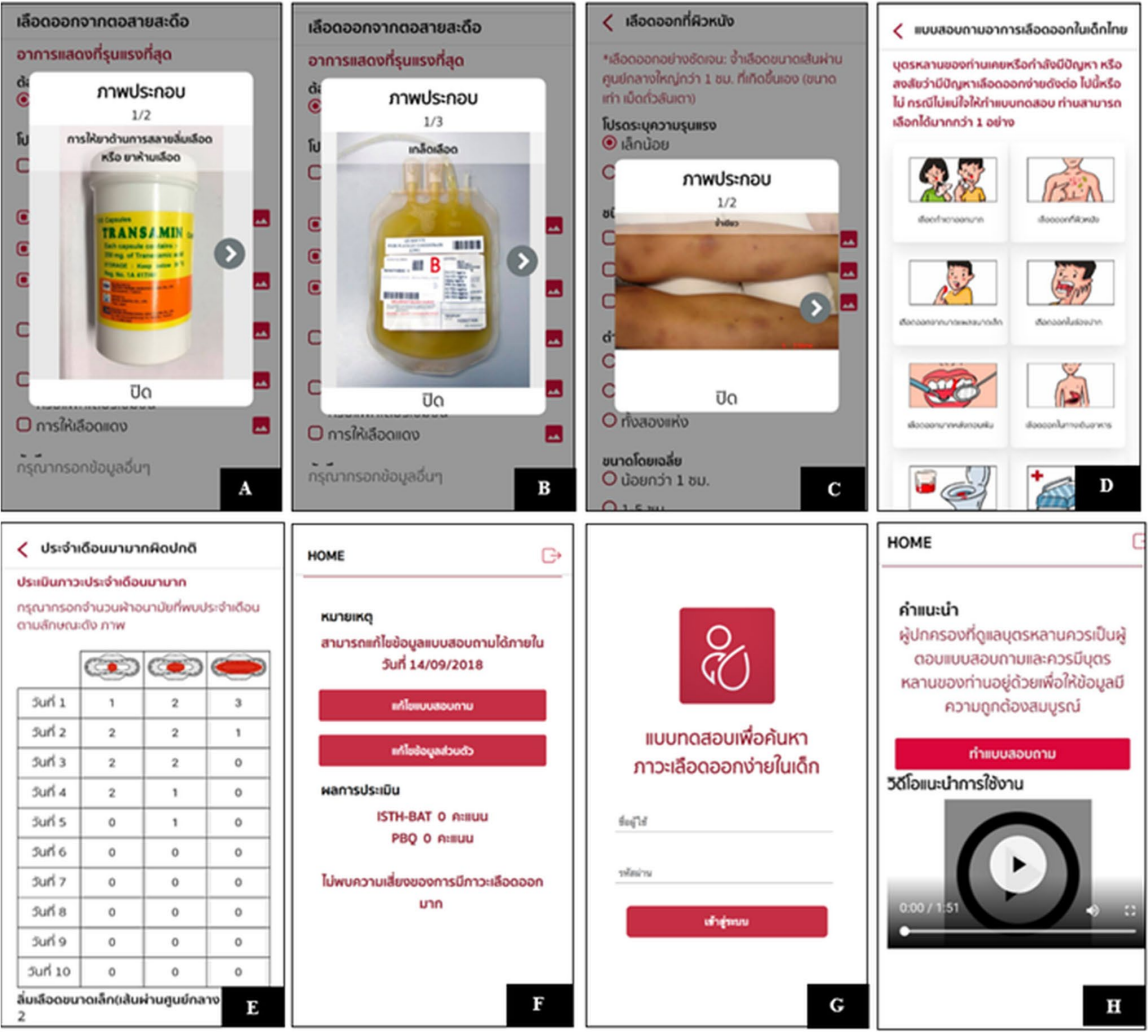
Pictorial illustration in the bleeding assessment tool mobile application (mBAT). (**A**), medication; (**B**), blood component; (**C**), cutaneous bleeding symptom; (**D**), the page includes 14 bleeding symptoms and no bleeding categories; (**E**), pictorial bleeding assessment chart; (**F**), calculated score; (**G**), login page; and (**H**), video illustration

The wording of the mBAT questionnaire was modified to make it more understandable for non-medical personnel by adding pictorial illustrations (Fig. 2A-2H). The mBAT questionnaire was divided into 14 items of bleeding with illustrating pictures, including menorrhagia, cutaneous bleeding, epistaxis, bleeding from minor wounds, oral cavity and after dental extraction, gastrointestinal bleeding, hematuria, postpartum hemorrhage, muscle hematomas, hemarthrosis, central nervous system (CNS) bleeding, and other bleedings (Fig. 2C, D). In females with heavy menstrual bleeding, mBAT was also linked to the pictorial bleeding assessment chart to automatically calculate the score (Fig. 2E). Furthermore, after completing the questionnaire, mBAT then calculated the total score with interpretation and uploaded it onto the ISTH-BAT and PBQ systems (Fig. 2F). The personal information was protected by a given unique password, which expired 72 hours after logging into the program (Fig. 2G). The scores and detailed information were collected on Excel. After developing the mBAT, the study team checked all contents of the questionnaire for accuracy and compared them with the TPBQ to confirm the similarity of the calculated scores. During the pre-study period, mBAT was applied to voluntary subjects and parents for their feedback, as a result, a short-video introduction on how to use mBAT was inserted at the beginning after user login to the system.

### Phase 2. Validation of the mBAT

**Fig. 3:**
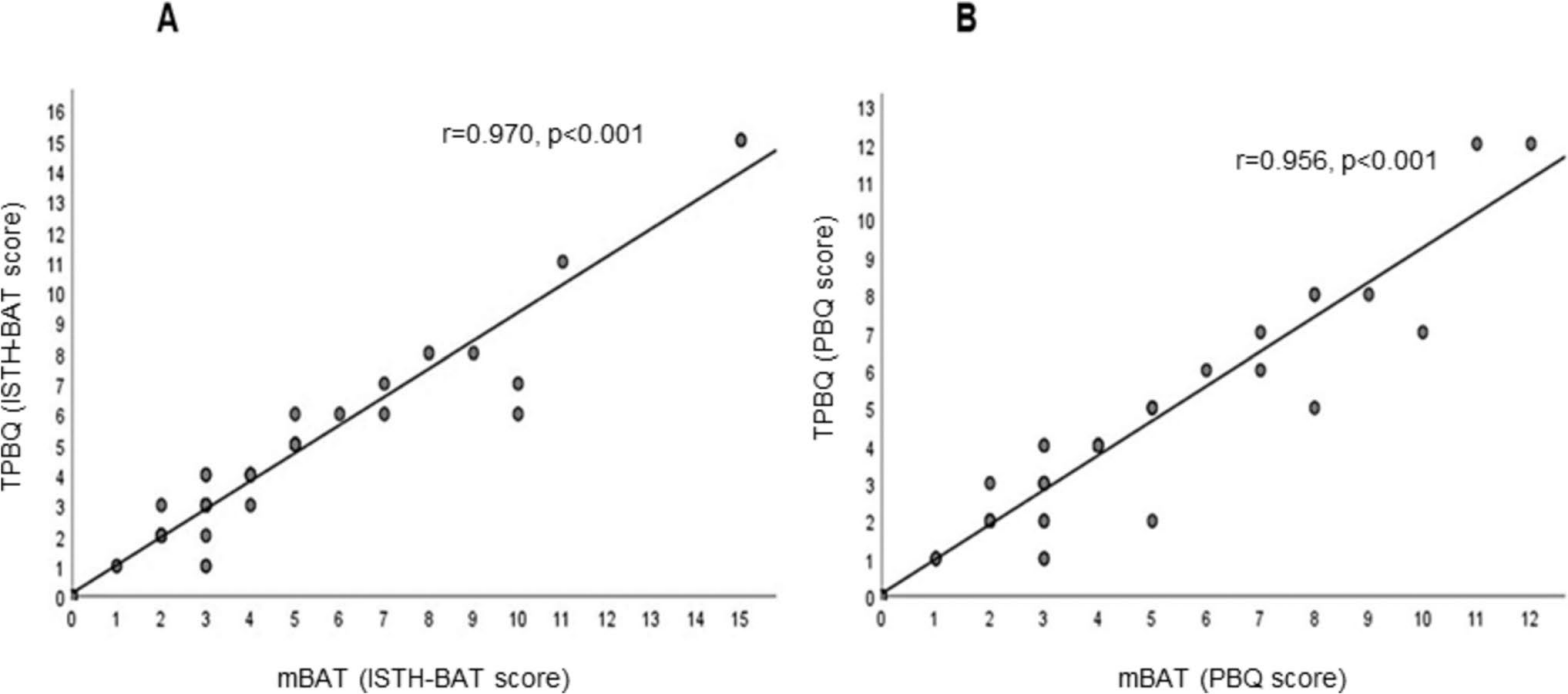
Scatter plots showing relation of the bleeding assessment tool mobile application (mBAT) and Thai version Pediatric Bleeding Questionnaire (TPBQ). A, PBQ scoring system; B, International Society on Thrombosis and Haemostasis Bleeding Assessment Tool (ISTH-BAT) scoring system

**Fig. 4:**
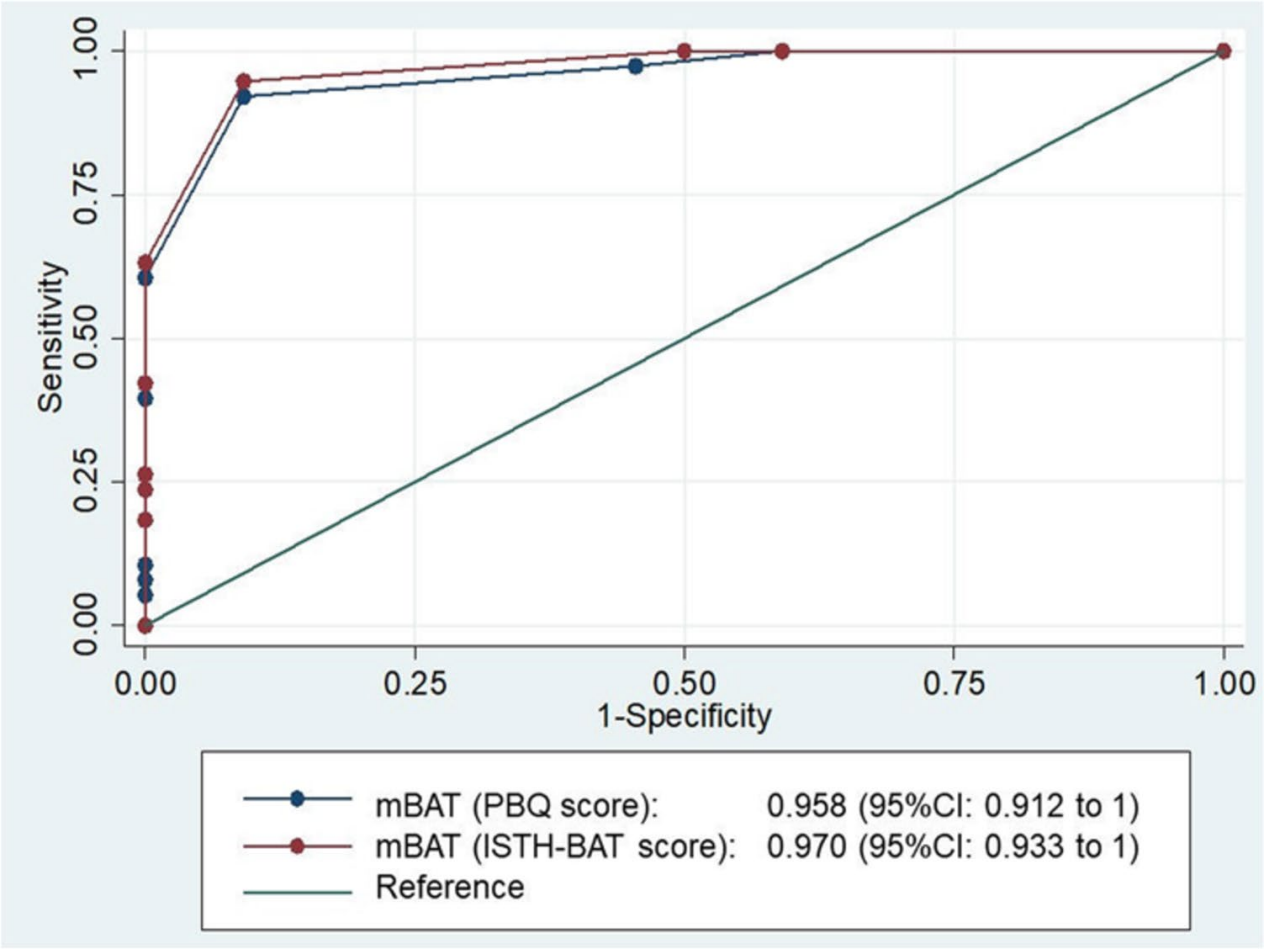
Area Under the Curve of bleeding assessment tool mobile application (mBAT) to predict the bleeding disorders by using cut-off score ≥3 for Pediatric Bleeding Questionnaire (PBQ) and International Society on Thrombosis and Haemostasis (ISTH-BAT) scoring systems of Thai version of Pediatric Bleeding Questionnaire (TPBQ)

Sixty subjects with known bleeding disorders were enrolled to validate the mBAT. Table 1 shows the bleeding disorders of the 60 subjects, including hemophilia A, hemophilia B, VWD type 1 and 2, platelet disorders, and other mild mucosal bleeding disorders. The female-to-male ratio was 1.7:1, and the mean (SD) and range for age was 10.5 (5.8) and 1–20.5 years, respectively. The mean (SD) and range interval between the subjects completing the mBAT and the paper-based TPBQ interviewed by medical personnel were 65 (2.2) and 35 to 70 days apart. The median (range) of overall bleeding scores from mBAT was 2 (0–15) from the ISTH-BAT and PBQ scoring systems. The highest score was identified in patients with Glanzmann thrombasthenia, followed by moderate and severe hemophilia, respectively. The scoring results were analyzed using the Pearson correlation coefficient method. The correlation coefficient (r) was 0.970 (*P*<0.001) in the ISTH-BAT scoring system, while the r of 0.956 (*P*<0.001) was observed in the PBQ system (Fig. 3). The results showed a strong correlation between the mBAT and the TPBQ when using bleeding scores ≥3 (Figs. 3A-B). Thus, a score of ≥3 was considered abnormal. The accuracies of PBQ and ISTH-BAT scores of ≥3 were 91.7% and 93.3%, respectively (*P*<0.001) (Table 2 and Fig. 4). According to the good correlation with TPBQ, our study decided to use a cut-off score of ≥3 to suggest abnormality. A satisfaction survey was reviewed by 32 randomly selected subjects and parents using the SUS method to evaluate the overall usability of an application. The SUS method was used with 10 questions with a 5-point Likert score. Then the SUS scale score was calculated, the result demonstrated an average score of 70. The interpretation was considered a good level of usability. To enhance the use of the application, therefore, a short-video introduction was inserted at the beginning after users logged into the system. The validated mBAT was submitted to the Department of Intellectual Properties, Ministry of Commerce, the Government of Thailand, and a patent # 389416 was issued.

**Table 1:** Characteristics of subjects for the validation of bleeding assessment tool mobile application (mBAT)

Baseline characteristic	Total	mBAT score [median (range)]		TPBQ Score [median (range)]	
	(N=60)	ISTH-BAT	PBQ	ISTH-BAT	PBQ
Gender [n (%)]					
Female	38 (63.3)	2 (0–15)	2 (0–11)	3 (0–15)	3 (0–12)
Male	22 (36.7)	2 (0–15)	2 (0–12)	3 (0–15)	3 (0–12)
Mean age-years (SD), and [range]	10.5 (5.8), [1–20.5]				
Diagnosis [n (%)]					
Hemophilia A	18 (30)				
-Mild hemophilia A	2 (3.3)	3.5 (2–5)	3.5 (2–5)	3.5 (2–5)	3.5 (2–5)
-Moderate hemophilia A	1(1.6)	6	3	6	3
-Severe hemophilia A	15 (25)	4 (0–15)	4 (0–12)	4 (0–15)	4 (0–12)
Hemophilia B	5 (8.3)				
-Moderate hemophilia B	1 (1.6)	3	3	3	4
-Severe hemophilia B	4 (6.7)	3 (0–7)	3 (0–7)	3.5 (0–6)	3.5 (0–6)
Von Willebrand disease	17 (28.3)				
-Von Willebrand disease type 1	12 (20)	3.5 (0–10)	3 (0–8)	3 (0–6)	3 (0–5)
-Von Willebrand disease type 2A	5 (8.3)	3 (2–7)	2 (1–7)	3 (2–7)	2 (1–7)
Platelet disorders	11 (18.3)				
-ADP receptor defect	3 (5)	3 (2–4)	3 (2–4)	2 (1–4)	2 (1–4)
-Glanzmann thrombasthenia	2 (3.3)	10 (5–15)	8.5 (6–11)	10.5 (6–15)	9 (6–12)
-Platelet dysfunction	1 (1.6)	3	3	2	2
-Congenital macrothrombocytopenia	2 (3.3)	2 (1–3)	2.5 (1–4)	2 (1–3)	2.5 (1–4)
-Platelet storage pool defect	2 (3.3)	2	2	2	2
-Wiskott-Aldrich syndrome	1 (1.6)	1	1	1	1
Other bleeding disorders	9 (15)				
-Afibrinogenemia	1 (1.6)	0	0	0	0
-Congenital factor VII deficiency	2 (3.3)	1.5 (0–3)	1.5 (0–3)	2 (0–4)	2 (0–4)
-Bleeding of unknown cause	6 (10)	2 (0–3)	3 (0–5)	2.5 (0–3)	2 (0–3)

Bleeding of unknown cause has been defined as a clear bleeding tendency in the presence of normal hemostatic tests, including complete blood count, partial thromboplastin time, prothrombin time, thrombin time, factor VIII:C, fibrinogen level, Von Willebrand factor antigen, Ristocetin cofactor activity, PFA-100, and platelet aggregation test. Abbreviation: ISTH-BAT, International Society on Thrombosis and Haemostasis Bleeding Assessment Tool; PBQ, Pediatric Bleeding Questionnaire

**Table 2:** Sensitivity, specificity, and accuracy of the cut-off bleeding score of bleeding assessment tool mobile application (mBAT) with Thai version of Pediatric Bleeding Questionnaire (TPBQ) and International Society on Thrombosis and Haemostasis Bleeding Assessment Tool (ISTH-BAT)

Scoring system of mBAT	Cut-off score	Sensitivity	Specificity	Accuracy	*P*-value
ISTH-BAT	1	100.0%	40.9%	78.3%	<0.001
	2	100.0%	50.0%	81.7%	<0.001
	3	94.7%	90.9%	93.3%	<0.001
PBQ	1	100.0%	40.9%	78.3%	<0.001
	2	94.7%	54.5%	81.7%	<0.001
	3	92.1%	90.6%	91.7%	<0.001

### Phase 3. Using the mobile application for self-screening in children

A total of 916 subjects, with a mean (SD) for age of 10.5 (4.7) years, and a female-to-male ratio of 3:2, were enrolled. As shown in Table 3, the majority (97.3%) of the subjects were from the community, and the rest (2.7%) were from the hematology clinic. The median (range) time to complete mBAT of patients with bleeding disorders after login to the application was 2.7 (1.03–5.63) minutes, while in subjects without bleeding disorders was 0.07 (0.02–46.2) minutes.

**Table 3:** Characteristics of 916 subjects for self-screening using Bleeding Assessment Tool Mobile Application (mBAT)

Parameter	Community	Hematology Clinic	Total
Number [n (%)]	891 (97.3)	25 (2.7)	916 (100)
Gender [n (%)]			
Male	355 (39.8)	12 (48)	367 (40.0)
Female	536 (60.2)	13 (52)	549 (60.0)
Mean age-years (SD), and [range]	10.5 (4.7), [1–18.1]	10.9 (5.1), [0.07–18]	10.5 (4.7), [0.07–18.1]
mBAT [Median score (range)]			
Pediatric Bleeding Questionnaire (PBQ)	0 (0–4)	3 (1–13)	0 (0–13)
International Society on Thrombosis and Haemostasis Bleeding Assessment Tool (ISTH-BAT)	0 (0–4)	3 (1–9)	0 (0–9)
Subjects with scores ≥3 [n (%)]	22 (2.5)	19 (76)	41 (4.5)
Subjects with scores ≥3, who consented for blood test	16 (1.8)	19 (76)	35 (3.8)
Subjects confirmed bleeding disorders [n (%)]	1 (6.25)	10 (52.6)	11 (31.4)
Bleeding assessment tool mobile application (mBAT)			

### Subjects in the community group

The mBAT was introduced to 1,742 children. The response rate of subjects who participated was 891 (51.1%). Children who participated in the community group included 891 subjects, which included 488 students ranging in age 12–18 years (grades 7–12) from four secondary schools and 403 children aged from 1 month to 18 years from well-child clinics. The mean (SD) and range for age were 10.5 (4.7) and 1–18.1 years, respectively. The median (range) of the mBAT scores according to the ISTH-BAT and the PBQ scoring systems were 0 (0–4) and 0 (0–4), respectively (Table 3). Twenty-two (2.5%) of the group had scores of ≥3, and 16 consented to blood testing and further investigation. From the test results, one subject (Patient 1, Table 4) was diagnosed with VWD type 1 and had heavy menstrual bleeding while the other 15 subjects had normal laboratory results. The diagnosis of VWD was referred to in the recent publication guidelines [19]. Thus, the prevalence of bleeding disorders in children with score of ≥3 in the community was 6.25% (Table 3).

**Table 4:** Characteristics of subjects from Phase 3 diagnosed with bleeding disorders

Patient number	Gender	Age (year)	Symptom	mBAT	PFA-100		APTT, PT, and TT (sec)	Fibrinogen (%)	VWF: Ag/VWF: RCo (%)	FVIII:C (%)	Platelet agg. test	Other laboratory testing	Diagnosis
				ISTH-BAT /PBQ	COL/EPI	COL/ADP							
1	F	12.6	Menorrhagia	3/3	170	124	Normal	216	46.7/36	135	Abnormal to Ristocetin	NA	VWD type 1
2	M	6.4	Epistaxis	4/4	197	161	Normal	225	33.2/36.8	48	Normal	NA	VWD type 1
3	F	13.8	Epistaxis	3/3	139	125	Normal	239	39.8/39.1	76.4	Normal	VWF multimer:normal	VWD type 1
4	M	10.9	Epistaxis	3/3	238	149	Normal	279	68.1/32	80.5	Abnormal to adrenaline	VWF multimer: normal *VWF* gene: c.4195C>T (p.Arg1399Cys), exon 28	VWD type 2M
5	F	5.2	Epistaxis	7/4	NA	NA	Normal	NA	NA	NA	NA	Platelet count: 27,000/cu mm Immunofluorescence to NMMHIIA: positive *MYH9* gene: c.2104>T (p.Arg702Cys), exon16	MYH9-related disorder
6	F	6.9	Epistaxis	4/5	NA	NA	Normal	NA	NA	NA	NA	Platelet count: 66,000/cu mm IPF: 40.3%, ANA: >1:1280 Flow cytometry: normal expression of CD41, CD61, CD42b NGS: normal	Thrombocytopenia suspected chronic ITP
7	F	17.9	Ecchymosis	4/2	NA	NA	Normal	252	72.1/71.3	82	Normal	Platelet count: 81,000/cu mm IPF: 7.3%, Flow cytometry: normal expression of CD41, CD61, CD42b	Thrombocytopenia suspected chronic ITP
8	M	3.2	Epistaxis	3/4	>283	>288	Normal	255	110/294	209	Abnormal to ADP 36.1% (impaired 2nd wave)	NA	ADP receptor defect
9	M	6.3	Ecchymosis	4/3	79	68	APTT 126	NA	128/137	0.4	Normal	*F8* gene: duplication in exon 24–25	Severe hemophilia A with alpha thalassemia disease, (hemoglobin H/ Constant Spring disease*)
10	M	13.6	Hematoma	13/13	97	77	APTT 91.5	395	NA	0.7	NA	FVIII Ab: 6.8 BU	Acquired hemophilia A from SLE
11	M	0.7	Conjunctival hemorrhage	4/4	NA	NA	PT 63.1, INR 6.19	NA	NA	NA	NA	FVII:C: 5.7% *F7* gene: homogenous of at c.1259G>T (p.Gly420Val), Exon 8	Factor VII deficiency

ADP, adenosine diphosphate; ANA, antinuclear antibody; APTT, activated partial thromboplastin time; COL/ADP, Collagen/Adenosine diphosphate; COL/EPI, Collagen/Epinephrine; F, female; FVII:C, factor VII clotting activity; FVIII:C, factor VIII clotting activity; FVIII Ab, factor VIII antibody; INR, international normalized ratio; IPF, immature platelet fraction; ISTH, International Society on Thrombosis and Haemostasis; ITP, Immune thrombocytopenia; M, male; mBAT, bleeding assessment tool mobile application; NA, not available; NGS, next generation sequencing; PBQ, Pediatric Bleeding Questionnaire; PFA, platelet function analysis; PT, prothrombin time; SLE, systemic lupus erythematosus; TT, thrombin time; VWD, Von Willebrand disease; VWF:Ag, Von Willebrand factor antigen; VWF:Activity, Von Willebrand factor activity

^*^α^CS^; HBA2 c.427T>C, --^SEA^; g.26264_45564 del 19

### Subjects in the hematology clinics group

The mean (SD) and range for age of the 25 subjects in the hematology clinics group was 10.9 (5.1) and 0.07–18 years, respectively. The median [range] of mBAT scores according to the ISTH-BAT and the PBQ scoring systems were 3 [1–9] and 3 [1–13], respectively (Table 3). Nineteen subjects of the group, who had scores ≥3, consented to laboratory testing and further investigation. Their answers regarding bleeding symptoms were also re-examined by physicians at the hematology clinic using TPBQ. Ten of the consented subjects were diagnosed with bleeding disorders, which included VWD type 1, and 2M, MYH9-related disorder, thrombocytopenia suspected chronic ITP, severe hemophilia A with underlying alpha-thalassemia disease, acquired severe hemophilia A from systemic lupus erythematosus, mild hemophilia A, ADP receptor defect, and factor VII deficiency. (Table 4). The diagnoses of bleeding disorders were based on their respective reported recommendations and/or guidelines [20–26], and the prevalence of the disorders with score ≥3 was 52.6%. For the other six subjects, whose scores were <3, their bleeding symptoms improved during the follow-up visits. Four of them were diagnosed with allergic rhinitis and the other two with dysfunction of uterine bleeding during menarche.

## Discussion

BATs have been used to screen for bleeding disorders in patients visiting hospitals with bleeding symptoms. However, some patients with unrecognized mild bleeding symptoms might not visit hospitals and have never been evaluated or investigated for bleeding disorders. The limited number of healthcare providers per population may also affect the diagnosis.

Screening bleeding disorders in the community was reported by using six simple bleeding symptoms as follows: 1) after surgery, 2) oral, 3) after mild trauma or injury, 4) heavy periods or postpartum, 5) skin, and 6) joint. For subjects who reported any abnormal bleeding symptoms, the standard ISTH-BAT was then performed. The positive screening subjects (33%) were further investigated. The results demonstrated an overall prevalence of bleeding disorders, including hemophilia, VWD, and immune thrombocytopenia, of 2.2/10,000 people, or 0.022% [27].

The first attempt to use a screening questionnaire for bleeding disorders in Thai children was reported by Laoaroon N. et al. The TPBQ, a paper-based Thai Pediatric-BAT, was applied to 309 subjects. Eight subjects had scores of ≥3, and they proceeded with further investigation, resulting in a diagnosis of VWD type 1 and VWD type 2A each in two subjects. The prevalence of bleeding disorders was equivalent to 0.65 in 100 subjects [13]. However, since the study required trained personnel to administer and was time-consuming, the TPBQ had limited use in large populations.

In recent years, a successful healthcare electronic BAT (eBAT) using REDCap® web-based software application was reported by Kaur et al. [14]. The results showed eBAT was an efficient tool, taking a shorter time to complete the questionnaire. The median duration (range) required time per patient to complete the eBAT was 8 (2–28) minutes, compared with 10 (3–19) minutes for paper-based BAT. A total of 47 patients (50%) had a specific bleeding diathesis diagnosis. The median eBAT score for those with a bleeding disorder was 5, with a strong positive correlation with the physician-administered bleeding questionnaire. The eBAT had a sensitivity of 93.8% (95% CI 82.8%–98.7%) and a specificity of 34.8% (95% CI 21.4%–50.3%) [14]. Our study supported the possibility of self-screening in the pediatric population. Apart from being a self-screening tool, the present study was the first to develop a pediatric BAT mobile application in the Thai language or mBAT. It can be easily installed on a smartphone or tablet from common iOS and Android digital platforms; therefore, the to-be screened subjects could concurrently complete the questionnaire from their mobile devices. Thus, it was suitable for large-scale screening. Additionally, the developmental process aimed to be user-friendly and easily understandable using visual aids such as pictures and video. It was convenient and time-saving to have a score automatically calculated with recommendations. Moreover, the study also measured subjects' and parents' satisfaction, which showed a good satisfaction score of usability. The reliability of this application was demonstrated by a good correlation with the paper-based Thai pediatric BAT in both PBQ and ISTH scoring systems. Therefore, PBQ or ISTH scoring systems could be implemented in the clinical setting. However, in the future, ISTH-BAT should be implemented to avoid confusion regarding the interpretation of the negative scoring items when used for self-screening without supervision from healthcare providers. The time to complete the screening was recorded through the application after a short-video introduction from the login to the score calculation. This demonstrated that subjects with bleeding disorders used more time than subjects without bleeding disorders per subject in the present study. The median (range) time was 2.7 (1.03–5.63) minutes and 0.07 (0.02–46.2) minutes, respectively. Compared with the previously reported completion time of paper-based BAT and eBAT, our mBATs were shorter [14]. However, there may be some limitations for time recording due to the internet connection or delayed logout to the application. In addition, the possible explanation for the short time recording was that the subjects could select “No bleeding symptoms” after reviewing the bleeding categories. (Fig. 2D).

Most screening subjects in our study were from communities. The authors enrolled subjects from the four schools. The prevalence of bleeding disorders in this report, calculated from subjects with a bleeding score of ≥3, was 6.25% (1/16). This report could not accurately determine the prevalence in the community because 27% (8/22) of the subjects with scores of ≥3 did not consent to a blood test. The prevalence of bleeding disorders in the Thai community using TPBQ by physicians was reported at 0.65% by Laoaroon et al. [13]. In that report, physicians introduced and explained the questionnaire face-to-face to the subjects, and all reported subjects with a score of ≥3 consented to further blood testing [13].

The authors also included subjects who visited the hospital with bleeding symptoms. Subjects with scores of ≥3 totaled 76%, which was higher than the community setting but almost similar to the previous report using eBAT in the REDCap® web-based software application [14]. After investigation, 52.6% (1/19) of the subjects with a score of ≥3 were diagnosed with bleeding disorders, which was almost similar to the previously reported 50% [14]. The most common diagnosis in the present study was VWD (36.3%), similar to what was reported in the previous study [14].

This study was faced with some challenges and limitations. One rather obvious challenge could be a recall bias of previous bleeding symptoms. Subjects might not recognize or remember bleeding symptoms when they were young. Secondly, the study enrolled 60 subjects with bleeding disorders without healthy control in the validation phase because the study took place during the COVID-19 pandemic; therefore, recruiting healthy children to participate in the research was strenuous due to school restrictions and the risk of contagion. Thirdly, not all subjects with a score of ≥3 were investigated. In total 16 (72.7%) subjects consented to blood testing and further investigation. Therefore, the prevalence of the disease could not be correctly calculated. In addition, there was a concern for most subjects (716 subjects, 78%) who chose 'no bleeding symptom' because they may not have followed the instructions to select any bleeding symptoms they experienced, resulting in a lower bleeding score. However, a previous study by Laoaroon et al. [13], which conducted in-person interviews with children using paper-based TPBQ, reported similar percentages to this study: 77% (144 out of 187 subjects) [13]. Fourthly, our study design did not include additional coagulation assays apart from factor VIII activity for all subjects with mBAT scores ≥3 and normal PT, aPTT, and TT. This could result in missed diagnoses of mild factor deficiencies, such as factor IX and factor XI deficiency. Fifthly, 875 subjects with a score of <3 were not investigated. Thus, the sensitivity and specificity of this mobile application could not be determined. Lastly, in Phase 3, the subjects used the mBAT, and only those with an mBAT score of ≥ 3 were re-evaluated using the TPBQ at hematology clinics, which confirmed the abnormal scores in those patients.

## Conclusions

mBAT was proven to have a strong correlation with the TPBQ. The mBAT process was simple, user-friendly, and less time-consuming as a self-screening tool. Furthermore, the self-screening mBAT was able to increase the diagnosis of bleeding disorders in the community or be included in the school health program. The mBAT may be of benefit in other settings, such as screening before elective surgery or procedure.

## Electronic Supplementary Material

Below is the link to the electronic supplementary material.

Supplementary Material 1 Supplementary Fig. 1 Thai language video illustration with translated English subtitle. (A-E), how to download and installation of mBAT; (F), log-in to the mBAT to begin the questionnaire; (G), suggestions to answer the questions with parent; (H), subjects can decide to fill the personal information; (I), suggestion to select a bleeding symptom, in hypermenorrhea, pictorial bleeding assessment chart appears after selecting this symptom; (J), pictures can be shown by clicking the icon of picture images; (K), suggestion to choose the bleeding symptoms; and (L), explain calculated score and result with suggestions

High Resolution Image (TIF 872 KB)

Supplementary Material 2 Sanger sequencing of affected gene in individual patients with bleeding disorders (A) Patient number 4, next-generation sequencing revealed a heterozygous in VWF gene, NM_000552.5:c.4195C>T (p.Agr1399Cys) in exon 28 and confirmed by sanger sequencing. (B) Patient number 5, Sanger sequencing of MYH9 gene in exon 16 revealed a heterozygous in MYH9 gene, NM_002473.6:c.2104>T (p.Arg702Cys). (C) Patient number 11, sanger sequencing of F7 gene in exon1-8 revealed a homozygous in FVII, NM_000131.4:c.1259G>T(p.Gly420Val) in exon 8. Parents had heterozygous of the same mutation. (D) Patient number 9, sanger sequencing of F8 gene did not identify the mutation. Further study with Multiplex ligation-dependent probe amplification (MLPA) result demonstrated duplication of exon 24-25 (red box) of F8.

High Resolution Image (TIF 21.3 MB)

## Data Availability

No datasets were generated or analysed during the current study.

## Abbreviations

APTT: Activated partial thromboplastin time
BATs: Bleeding assessment tools
CBC: Complete blood count
CNS: Central nervous system
eBAT: Electronic self-administered bleeding assessment tool
ISTH-BAT: International Society on Thrombosis and Haemostasis Bleeding Assessment Tool
ITP: Immune thrombocytopenia
mBAT: Bleeding assessment tool mobile application
PBQ: Pediatric Bleeding Questionnaire
PFA-100: Platelet function analysis-100
PT: Prothrombin time
SUS: System usability scale
TPBQ: Thai version of Pediatric Bleeding Questionnaire
TT: Thrombin time
VWD: Von Willebrand disease
WFH: World Federation of Hemophilia
