# Supplementary figures and images for: mBAT: a newly developed mobile application for self-screening of pediatric bleeding disorders – a multi-center study
Source: Ann Hematol. 2025 Jan 15;104(1):131–43. doi: 10.1007/s00277-024-06178-w (PMC11868147; doi:10.1007/s00277-024-06178-w)

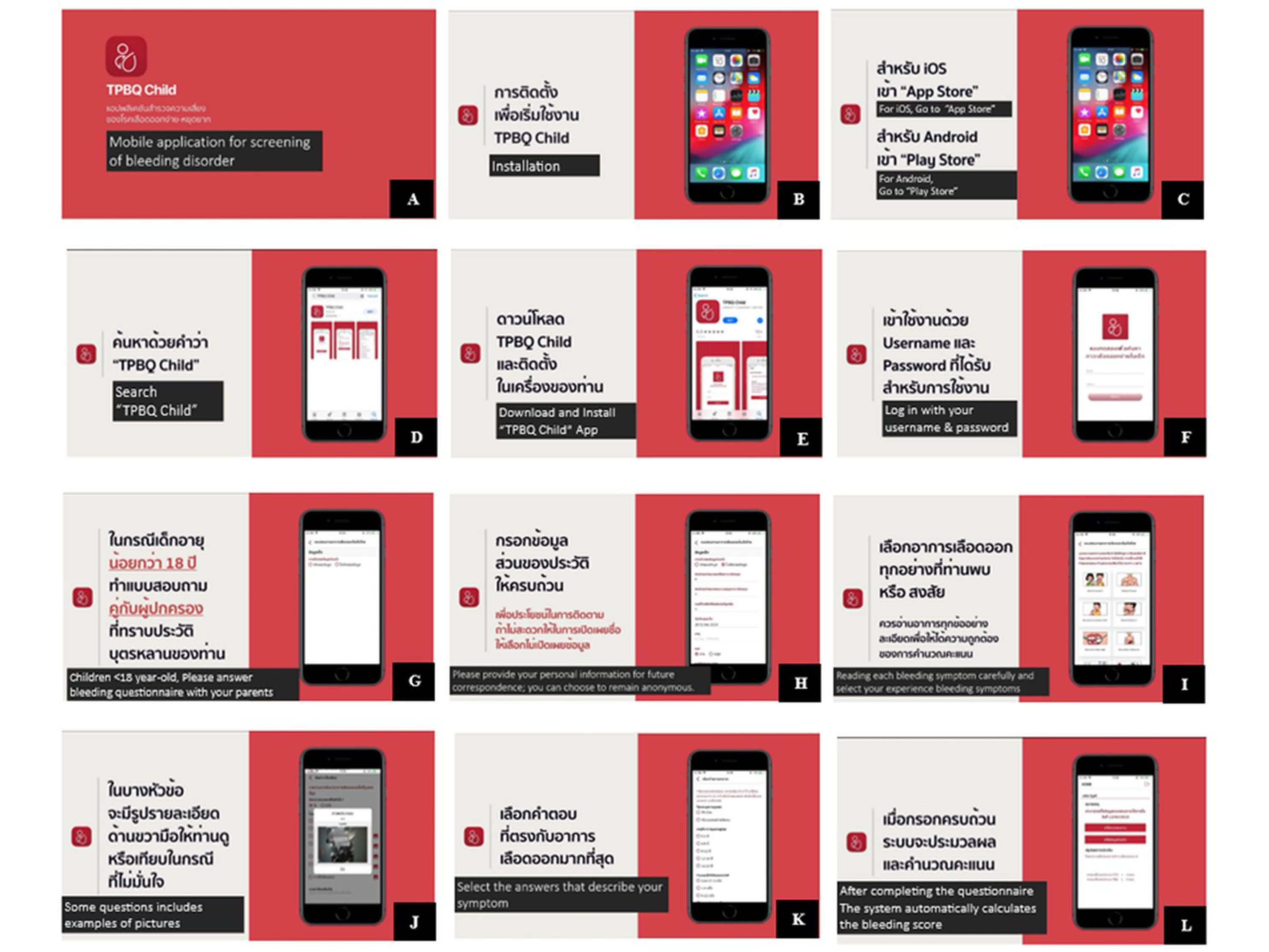

Supplement: Supplementary file 1 — Supplementary Material 1 Supplementary Fig. 1 Thai language video illustration with translated English subtitle. (A-E), how to download and installation of mBAT; (F), log-in to the mBAT to begin the questionnaire; (G), suggestions to answer the questions with parent; (H), subjects can decide to fill the personal information; (I), suggestion to select a bleeding symptom, in hypermenorrhea, pictorial bleeding assessment chart appears after selecting this symptom; (J), pictures can be shown by clicking the icon of picture images; (K), suggestion to choose the bleeding symptoms; and (L), explain calculated score and result with suggestions [file 277_2024_6178_Fig8_ESM.png]

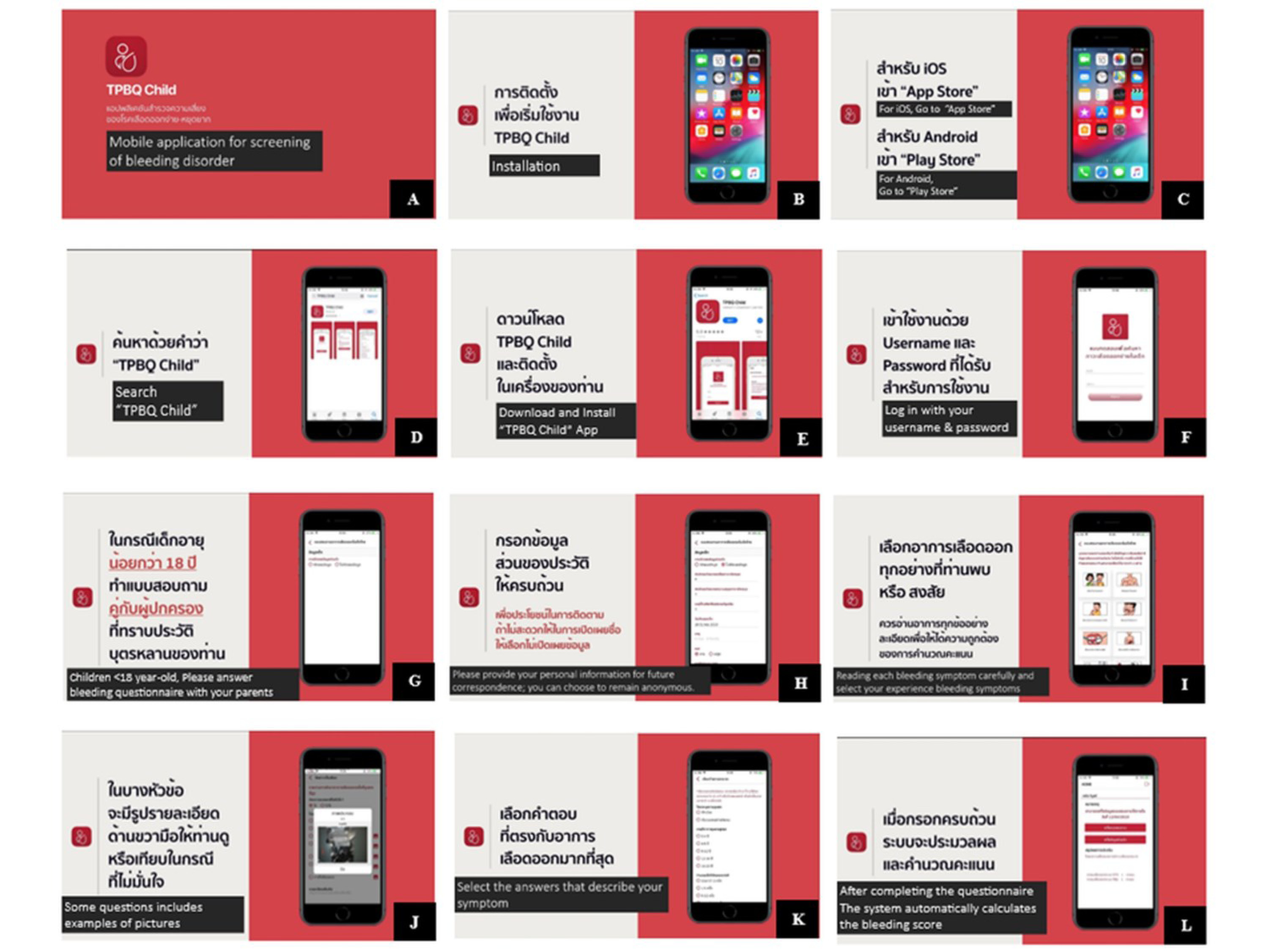

Supplement: Supplementary file 2 — High Resolution Image (TIF 872 KB) [file 277_2024_6178_MOESM1_ESM.tif]

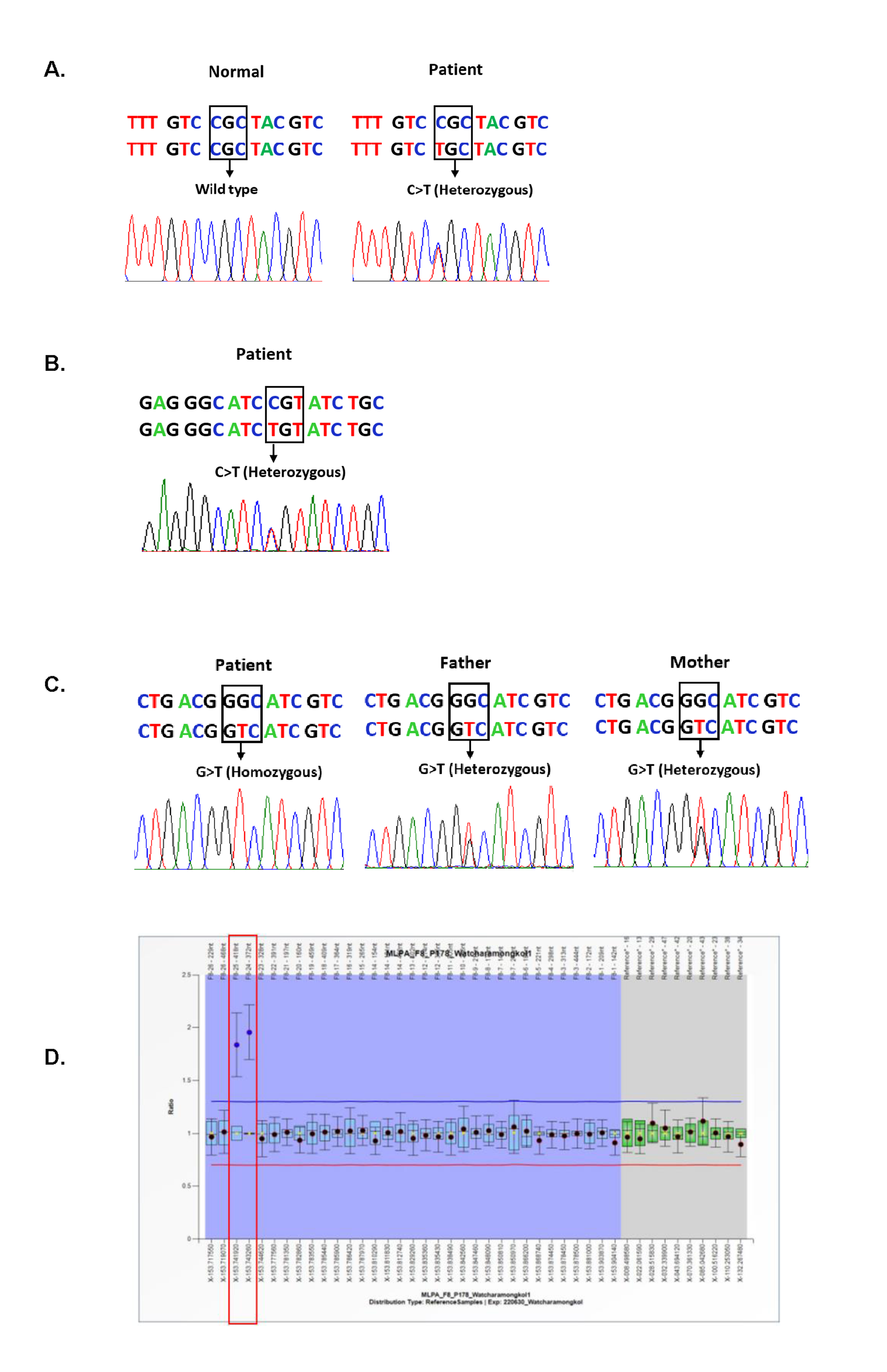

Supplement: Supplementary file 3 — Supplementary Material 2 Sanger sequencing of affected gene in individual patients with bleeding disorders (A) Patient number 4, next-generation sequencing revealed a heterozygous in VWF gene, NM_000552.5:c.4195C>T (p.Agr1399Cys) in exon 28 and confirmed by sanger sequencing. (B) Patient number 5, Sanger sequencing of MYH9 gene in exon 16 revealed a heterozygous in MYH9 gene, NM_002473.6:c.2104>T (p.Arg702Cys). (C) Patient number 11, sanger sequencing of F7 gene in exon1-8 revealed a homozygous in FVII, NM_000131.4:c.1259G>T(p.Gly420Val) in exon 8. Parents had heterozygous of the same mutation. (D) Patient number 9, sanger sequencing of F8 gene did not identify the mutation. Further study with Multiplex ligation-dependent probe amplification (MLPA) result demonstrated duplication of exon 24-25 (red box) of F8. [file 277_2024_6178_Fig9_ESM.png]

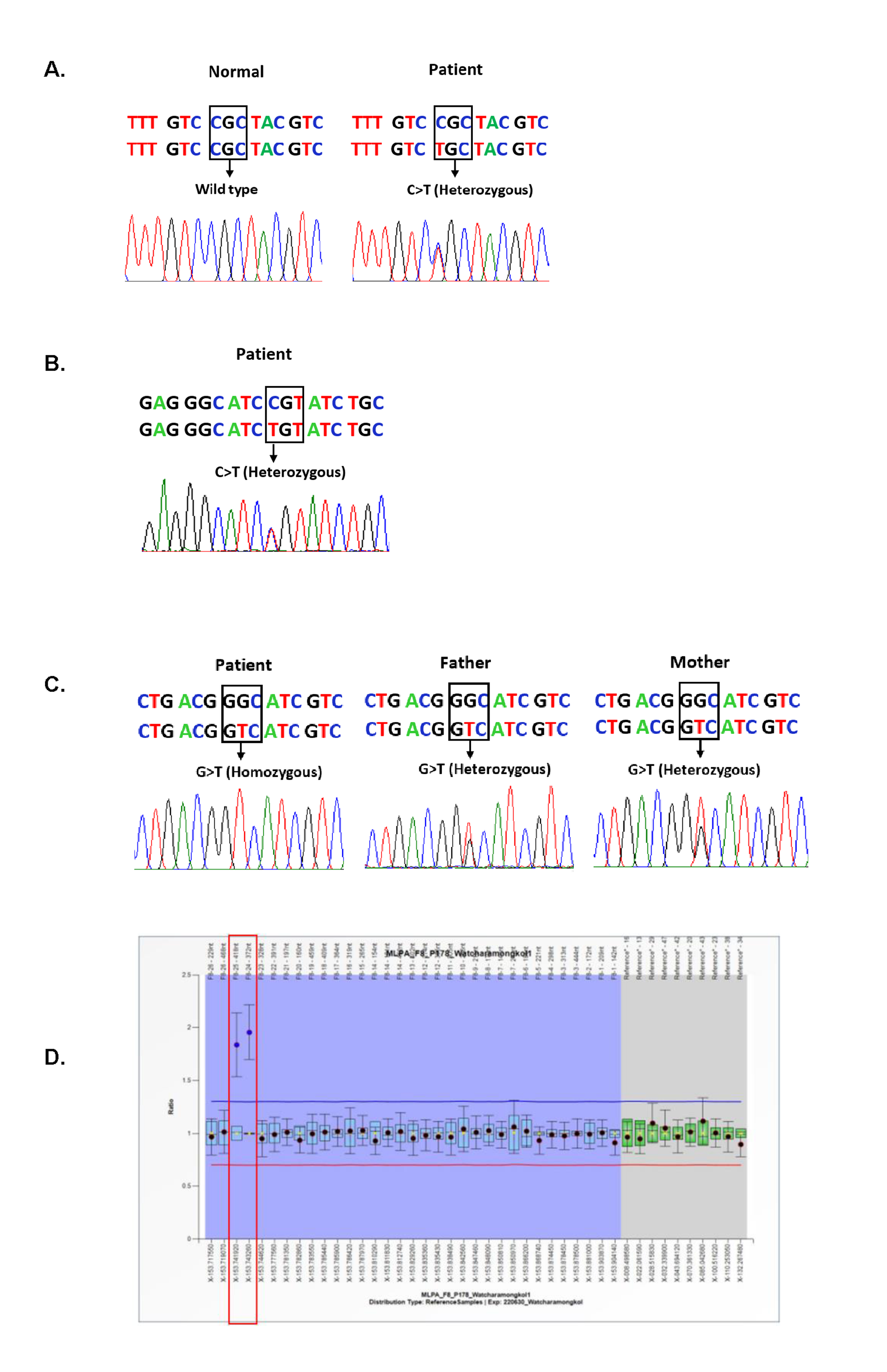

Supplement: Supplementary file 4 — High Resolution Image (TIF 21.3 MB) [file 277_2024_6178_MOESM2_ESM.tif]
